# A Multipopulation PSO Based Memetic Algorithm for Permutation Flow Shop Scheduling

**DOI:** 10.1155/2013/387194

**Published:** 2013-12-15

**Authors:** Ruochen Liu, Chenlin Ma, Wenping Ma, Yangyang Li

**Affiliations:** Key Laboratory of Intelligent Perception and Image Understanding of Ministry of Education of China, Xidian University, Xi'an 710071, China

## Abstract

The permutation flow shop scheduling problem (PFSSP) is part of production scheduling, which belongs to the hardest combinatorial optimization problem. In this paper, a multipopulation particle swarm optimization (PSO) based memetic algorithm (MPSOMA) is proposed in this paper. In the proposed algorithm, the whole particle swarm population is divided into three subpopulations in which each particle evolves itself by the standard PSO and then updates each subpopulation by using different local search schemes such as variable neighborhood search (VNS) and individual improvement scheme (IIS). Then, the best particle of each subpopulation is selected to construct a probabilistic model by using estimation of distribution algorithm (EDA) and three particles are sampled from the probabilistic model to update the worst individual in each subpopulation. The best particle in the entire particle swarm is used to update the global optimal solution. The proposed MPSOMA is compared with two recently proposed algorithms, namely, PSO based memetic algorithm (PSOMA) and hybrid particle swarm optimization with estimation of distribution algorithm (PSOEDA), on 29 well-known PFFSPs taken from OR-library, and the experimental results show that it is an effective approach for the PFFSP.

## 1. Introduction

As a well-known problem in the area of scheduling, PFSSP is defined as the processing sequence of all jobs is the same for all machines, for minimizing the maximum completion time (i.e., makespan) [1]. Concerning its complexity, PFSSP has been proved to be a combinatorial optimization problem and a nondeterministic polynomial-time (NP) hard combinatorial optimization problem. Nevertheless, it is important to develop effective and efficient approaches for PFSSP due to its significance in theory and practical engineering application.

During the past decades, many heuristic methods have been introduced for solving PFSSP, and they can be classified into three categories: constructive heuristic methods, improved heuristic methods, and hybrid heuristic algorithm [2]. The algorithms proposed in [3–5] belong to the constructive heuristic methods. These methods get the solutions by rules defined in advance, but the rules are not suitable for every environment. Then, some researchers made use of improved heuristic to get the solutions, including simulated annealing algorithm [6], Tabu search method [7], genetic algorithm [8, 9], and particle swarm optimization algorithm [10]. But these algorithms get the local optimal value at the most time and have some other limitations. However, these algorithms suffer from time-consuming and parameter dependent. Hybrid heuristic algorithms also called as memetic algorithms (MA) can be considered as a union of a population-based global search and local improvements and result in more robust and effective optimization tools. It is well known that the performances of global search algorithms such as evolutionary algorithm and PSO can be improved by combining with problem-dependent local searches. For PFSSP, many hybrid heuristic algorithms have also been proposed. In [11], simulated annealing (SA) and genetic algorithm-(GA-) based hybrid heuristic algorithms were proposed by incorporating a modified Nawaz-Enscore-Ham (NEH) [12] heuristic. In [13], PSO algorithm combined with the variable neighborhood-based local search algorithm is proposed. In [2], an efficient particle swarm optimization based mimetic algorithm (MA) for PFSSP is proposed. In [14], a discrete particle swarm optimization (DPSO) algorithm is proposed to solve permutation flow shop scheduling problems, which use a local search procedure to escape the algorithm from the local optima. Reference [15] presented a review and comparative evaluation of heuristics and metaheuristics for the permutation flow shop problem with the makespan criterion. A comparison of 25 methods, ranging from the classical Johnson's algorithm (dispatching rules) to the recent metaheuristics, was proposed in the paper. In addition, an evaluation composed of 120 instances of different sizes was introduced as well. Reference [16] introduced a similar particle swarm optimization algorithm (SPSOA) applied for permutation flow shop scheduling to minimize makespan, which is based on the improvement of the option modes of the global optimal solution and the local optimal solution in PSO. In [17], a novel particles warm optimization (NPSO) algorithm was presented and successfully applied to permutation flow shop scheduling. In [18], a hybrid simulated annealing algorithm is proposed for the job shop scheduling problem with the objective of minimizing total weighted tardiness. The proposed algorithm was based on a novel immune mechanism whose fundamental idea was as follows: the bottleneck jobs existing in each scheduling instance generally constitute the key factors in the attempt to improve the quality of final schedules, and, thus, the sequencing of these jobs needs more intensive optimization.

In this paper, we propose a multipopulation PSO-based MA algorithm. The novelty of the algorithm lies in several aspects. First, multipopulation scheme is used to increase the diversity of the population. In the proposed algorithm, the whole population is divided into three subpopulations in which each particle evolves itself by standard PSO and then updates its best position with different local search schemes. Secondly, the cooperation among the subpopulations is achieved by an estimation of distribution algorithm (EDA) [19]. The best particle of each subpopulation is selected to construct a probabilistic model which characterizes the distribution of promising solutions in the search space in each generation. Third, to make the standard PSO suit for the PFSSP, a ranked-order value (ROV) [20] rule is presented to convert the continuous position of particles to permutation of jobs.

The rest of this paper is organized as follows. In Section 2, some related backgrounds about PFSSP and the standard PSO are introduced, respectively. In Section 3, the framework of MPSOMA algorithm is proposed, and solution representations, multipopulation cooperative coevolution, and local search schemes are introduced in detail. In Section 4, 29 well-known PFFSPs taken from OR-library are used to evaluate the proposed algorithm. Finally, summary and conclusions are drawn in Section 5.

## 2. Related Background

### 2.1. PFSSP

PFSSP can be stated as follows: there are *n* jobs to be scheduled with known processing time on *m* machines. At one time, each job can be processed on one machine at most and each machine can process one job at most. All of the jobs should be processed with the same permutation in all machines. The objective of PFSSP is to find an appropriate permutation schedule for jobs that minimizes the maximum completion time.

Let *π* = {*j*_1_, *j*_2_,…, *j*_*n*_} be a permutation of all jobs, let *p*_*i*,*j*_ be the process time of the job *i* on the machine *j*, and let *C*(*j*_*i*_, *k*) be the completion time of job *j*_*i*_ on machine *k*; the completion time *C*(*j*_*i*_, *k*) can be calculated as follows:

(1) $$\begin{array}{c} C\left(j_{1},1\right)=p_{j_{1},1}, \end{array}$$

(2) C(ji,1)=C(jj−1,1)+pji,1, i=2,…,n,

(3) C(j1,k)=C(j1,k−1)+pj1,1, k=2,…,m,

(4) C(ji,k)=max⁡{C(ji−1,k),C(ji,k−1)}+pji,k,i=2,…,n, k=2,…,m,

(5) $$\begin{array}{c} C_{\max}\left(\pi \right)=C\left(j_{n},m\right). \end{array}$$

The PFSSP is to find a permutation π* in the set of all permutation Π, such that

(6) Cmax⁡(π∗)≤C(πn,m) ∀π∈Π.

### 2.2. Standard PSO Algorithm

PSO is inspired by the social interaction behavior of birds flocking and fish schooling [21]. To search for the optimal solution, each bird, which is typically called a “particle,” updates its flying velocity and current position iteratively according to its own flying experience and other “particles” flying experience. At each iteration, the velocity vector for each particle is modified based on three parameters: the particle momentum, the best position reached by the particle, and that of all particles up to the current stage. Then, according to the determined velocity for each particle, the particle is moved to its next position. In PSO, a population of conceptual “particle” is initialized with random positions *X*_*i*_ and velocities *V*_*i*_, and a function *f* is evaluated by using the particle's positional coordinates as input values. In an *n*-dimensional search space, *X*_*i*_ = (*X*_*i*1_, *X*_*i*2_,…, *X*_*id*_) and *V*_*i*_ = (*V*_*i*1_, *V*_*i*2_,…, *V*_*id*_). Positions and velocities are adjusted, and the function is evaluated with the new coordinates at each time step. The basic update equations for the *d*th dimension of the *i*th particle in PSO are expressed in (7):

(7) xi,j(t+1)=xi,j(t)+vi,j(t+1) j=1,…,d,

(8) vi,j(t+1)=wvi,j(t)i,j+c1r1(pbest,j−xi,j(t))+c2r2(gbest,j−x(t))j=1,…,d,

where *w* is the inertia weight that controls the impact of previous velocity of particle on its current one. *c*_1_ and *c*_2_ are positive constant parameters called acceleration coefficients which control the maximum step size between successive iterations. *r*_1_ and *r*_2_ are two independently uniformly distributed random variables in range [0,1]. *P*_best_ = [*p*_best,1_, *p*_best,2_,…, *p*_best,*d*_] is the local best solution found so far by the *i*th particle, while *g*_best_ = [*g*_best,1_, *p*_best,2_,…, *p*_best,*d*_] represents the positional coordinates of the fittest particle found so far in the entire community or in some neighborhood of the current particle. Once the iterations are terminated, most of the particles are expected to converge to a small radius surrounding the global optima of the search space. There are two common conditions used for terminating the iterative process. PSO stops when it exceeds the predefined number of iterations, or there is negligible change for particles in successive iterations.

Similar to genetic algorithms, PSO is also a population-based iterative algorithm. But PSO requires less computational bookkeeping and generally fewer lines of code. Unlike the GA, PSO does not have complicated evolutionary operators such as crossover and mutation. The process of the standard PSO is shown in Algorithm 1. 

## 3. Multipopulation PSO-MA

The multipopulation PSO-MA algorithm is proposed in this section; framework its described in Algorithm 2. First, generate an initial swarm and divide it into three subpopulations evenly. Particle in each subpopulation updates its velocities and positions by using (7). And then two local searches are applied to all particles of three subpopulations with different probabilities. (IIS Individual improvement scheme) local search is applied on subpopulation1 and subpopulation3, while VNS (variable neighborhood search) local search is used to update subpopulation2. The best particle of each subpopulation is chosen to construct the EDA probabilistic model. From the probabilistic model, we sample three new particles which are used to update the worst individuals of the corresponding subpopulation. Then, the best individual is selected from all individuals of three subpopulations to be performed (SA simulated annealing). Finally, *g*_best_^  ^  is updated.

### 3.1. Solution Representation and Population Initialization

One of the key issues when designing the PSO algorithm is its solution representation. PFSSP is set in a discrete space. It is obvious that standard PSO equations cannot be used to generate a discrete job permutation since positions and velocities are real-valued. So, the most important issue in applying PSO to PFSSP is to develop a suitable mapping between positions of particles and job sequence.

In this paper, a ROV rule [20] based on random key representation is used to convert the continuous position *X*_*i*_ = [*x*_*i*,1_, *x*_*i*,2_,…, *x*_*i*,*n*_] of particles in PSO to permutation of jobs *π* = {*j*_1_, *j*_2_,…, *j*_*n*_}; thus, the performance of the particle can be evaluated. In our ROV rule, the position information *X*_*i*_ = [*x*_*i*,1_, *x*_*i*,2_,…, *x*_*i*,*n*_] itself does not represent a sequence, whereas the rank of each position value of a particle represents a job index so as to construct a permutation of jobs. The smallest position value of a particle is assigned rank 1. Then, the second smallest position value is assigned to rank 2. With the same way, all the position values will be handled to convert the position information of a particle to a job permutation. We provide a simple example to illustrate the ROV rule in Table 1. In the beginning, position is *x*_*i*_ = [0.6,2.9,1.8,3.7,2.1,0.7]. Because *x*_*i*,1_ = 0.6 is the smallest position value, *x*_*i*,1_ is picked first and assigned rank value 1; then, *x*_*i*,6_ = 0.7 is picked and assigned rank value 2. Similarly, the ROV rule assigns rank values 3 to 6 to *x*_*i*,3_, *x*_*i*,5_, *x*_*i*,2_, and *x*_*i*,4_, respectively. Thus, based on the ROV rule, the job permutation is obtained *π* = {1,5, 3,6, 4,2}.

In the standard PSO, each particle is initialized in a real space randomly, and an initial swarm is composed by a set of particles. In this paper, we use the same method as [2] to generate the initial swarm. A solution (i.e., permutation of jobs) is generated by using NEH heuristic [12], and the rest particles are initialized as the standard PSO; namely, they are generated with a random position values and velocities in a certain interval.

The results produced by NEH heuristic or the local search are job permutations, so they should be converted to the position values of a certain particle to perform PSO based searching. The conversation is performed using the following equation [22]:

(9) xNEH,j=xmin⁡,j+(xmax⁡,j−xmin⁡,j)(sNEH,j−1+rand)n,j=1,2,…,n,

where *x*_NEH,*j*_ is the position value in the *j*th dimension of the particle, *s*_NEH,*j*_ is the job index in the *j*th dimension of the permutation by the NEH heuristic, *x*_max⁡,*j*_ and *x*_min⁡,*j*_ are the upper and lower bounds of the position value, respectively, rand denotes a random number uniformly distributed in the interval [0, 1], and *n* represents the number of dimensions of a position, which is equal to the number of jobs.

### 3.2. EDA for PFSSP

In the proposed algorithm, we divide the whole population into three subpopulations. The relation between the subpopulation is not competitive but cooperative coevolution. Cooperative coevolution [23] is inspired by the ecological relationship of symbiosis where different species live together in a mutually beneficial relationship. The basic idea of cooperative coevolution is to divide and conquer: divide a large system into many modules, evolve the modules separately, and then combine them together to form the whole system. In this paper, we divide the population into three subpopulations; firstly, each of these subpopulations is evolved by using the standard PSO and then updated subpopulations are optimized by local search schemes further. Then, the information of best individual of each subpopulation is shared by EDA [24].

EDAs were firstly introduced in [19] which is a class of novel population-based evolutionary algorithms. Unlike traditional evolutionary algorithms, new solutions are sampled from EDA probabilistic model which characterizes the distribution of promising solutions in the search space at each generation. Due to its effectiveness and search ability, EDA has recently attracted much attention in the field of evolutionary computation, and it has already been applied to solve combinatorial optimization problems, including the flow shop scheduling problem.

In our proposed algorithm, we select three individuals to construct a probabilistic model. In every generation, particle in each subpopulation evolves with the standard PSO operation and then updates its best position by using two different local search schemes. The best particle of each subpopulation is chosen and then decoded to the permutation of the job. Instead of using the conventional crossover and mutation operations in evolutionary algorithm, EDA estimates a probabilistic model from the information of the selected three individuals in the current generation, which is represented with a conditional probability distribution for each decision variable. Here, we will introduce the process simply.

Let three sequences of jobs corresponding to three best particles selected from three subpopulations after the local search be *π*_1best_, *π*_2best_, and *π*_3best_, let *η*_*jk*_ be the number of times of appearance of job *j* before or in the position *k* in the subset of the selected sequences augmented by a given constant *δ*_1_, and let *μ*_*j*[*k*−1]_ be the number of times of appearance of job *j* immediately after the job in the position *k* − 1 in the subset of the selected sequences augmented by a given constant *δ*_2_. Here, The value of *η*_*jk*_ refers to the importance of the order of the jobs in the sequence and *μ*_*j*[*k*−1]_ refers to the importance of the similar blocks of jobs in the sequences.

We define *ρ*(*k*, *j*) the probability of selection of the job *j* in the *k*th position by the following formula:

(10) $$\begin{array}{c} \rho \left(k,j\right)=\frac{\eta_{jk}\times \mu_{j[k-1]}}{\sum_{l\in \Omega_{k}}\left(\eta_{lk}\times \mu_{l[k-1]}\right)}, \end{array}$$

where *Ω*_*k*_ is the set of jobs not already scheduled until position *k*. According to this probability, for each position *k*, we select a job *j* from the set of not already scheduled jobs in the sequence of a new individual.

In the proposed algorithm, three new sequences of jobs *π*_1_, *π*_2_, and *π*_3_ are sampled from the probabilistic model. We compare *π*_1_, *π*_2_, and *π*_3_ with the corresponding three worst individuals *π*_1wor_, *π*_2wor_, and *π*_3wor_ in the current swarm. If *π*_*i*_ is better than *π*_*i*wor_ and *π*_*i*_ is unique if compared with those individuals in the current subpopulation, then, *π*_*i*wor_ is removed from the swarm and replaced with *π*_*i*_.

### 3.3. IIS Local Search

In this section, a local search named individual improvement scheme (IIS) [25] is introduced. It is performed on each corresponding sequence of the job decoded by each particle in subpopulation1 and subpopulation3. The procedure of IIS local search is shown in Algorithm 3.

IIS examines each possible pairwise interchange of the job in first position, with the jobs in all other positions. Similarly, the jobs in the second and other subsequent positions are examined. Whenever there is an improvement in the objective function, the new individual is accepted. IIS local search can be viewed as a detailed neighborhood searching process, which can improve the individuals with a certain probability.

### 3.4. VNS Local Search

variable neighborhood search [26], a metaheuristic proposed just a few years ago, is based upon a simple principle: systematic change of neighborhood within the search. It combines efficient local optimization procedures with the heuristics which can escape from the local optima by restructuring the neighborhood. Different neighborhood provide different candidate solutions; thus, it is possible to find out a better solution.

In this paper, the particles are decoded to sequences of jobs of subpopulation2. The VNS local search is applied to all job sequences with a certain probability. In VNS, two structures of neighborhood are adopted which are called swap and insert.

*Swap.* Select two distinct elements from a job permutation and swap them.

*Insert.* Choose two distinct elements from a job permutation randomly and insert the back one before the front one.

The procedure of VNS is shown in Algorithm 4. The method described above is applied to all the particles of subpopulation3 with probability at each generation.

The first operation leads to all possible swaps of pairs of job's positions, within all parts of solutions. If the swap moves are performed, the second structure of neighborhood consists of all possible insert moves of pairs of positions of jobs, within all parts of the so obtained solution. Then, we return to the swap with the improved solution as a current solution. We reapply this procedure until the stopping condition.

### 3.5. SA Local Search

SA is a neighborhood search technique which can produce good results for combinatorial problems. A standard SA procedure begins by generating an initial solution at random. At each stage, the new solution taken from the neighborhood of the current solution is accepted as the new current solution if it has a lower or equal cost; if it has a higher cost, it is accepted with a probability.

In this paper, a SA-based local search with multiple different neighborhoods is developed to enrich the local searching behaviors and avoid premature convergence. In SA, two different kinds of neighborhood are used. The SA algorithm randomly generates a new state in the neighborhood of the original one, which causes a change of Δ*E* in the objective function value. For minimization problems, the new state is accepted with probability min⁡  {1, exp⁡(−Δ*E*/*T*)}, where *T* is a control parameter. SA provides a mechanism to probabilistically escape from local optima, and the search process can be controlled by the cooling schedule. The SA-based local search is only applied to *g*_best_.  Algorithm 5 shows the process of SA.

## 4. Experimental Results

In this section, 29 well-studied benchmarks problems which were contributed to the OR-library are selected to evaluate the performance of the proposed algorithm. The first eight problems are car1, car2, through car8 provided by Carlier [27]. The other 18 problems are called Rec01–Rec41 given by Reeves [8]. So far, these problems have been used as benchmarks for study with different methods by many researchers.

The following parameters are used in algorithm: the size of swarm *ps* = 3∗*n* (*n* is the number of the jobs); *w* = 1.0, *c*_1_ = *c*_2_ = 2.0, *x*_min⁡_ = −4.0, *x*_max⁡_ = 4.0, *v*_min⁡_ = −4.0, and *v*_max⁡_ = 4.0; the probability of IIS local search: *P*_LIS_ = 0.5; the probability of VNS local search: *P*_VNS_ = 0.2; the initial temperature *T*_0_ = 3; the annealing cooling rate *d* = 0.9; and the maximum generation is 150. Each instance is independently run 20 times for every algorithm for comparison. All parameters in the proposed algorithm are set as the compared algorithms in order to make a fair comparison; only the size of swarm is set differently from the compared algorithms since three subpopulations are employed in the MPSOMA.

In Sections 4.1 and 4.2, we will compare the performance of the proposed algorithm with those of two state-of-art PSO algorithms PSOMA [2] and PSOEDA [22]. Then, the usefulness of local search will be discussed in Section 4.3.

The proposed algorithm is implemented in MATLAB 7.0 on a personal computer with 2.4-GHz Inter Core2 Duo CPU and 2-GB RAM and MATLAB 7.0 programming tools.

### 4.1. Comparisons of PSOMA and MPSOMA

To evaluate the effectiveness of multipopulation scheme in the proposed algorithm, we first compare the proposed algorithm with an effective PSO-based memetic algorithm (PSOMA) proposed by Bo Liu et al. [2]. In the PSOMA, both PSO-based searching operators and some special local searching operators are designed to balance the exploration and exploitation. SA-based local search with multiple different neighborhoods is also designed and incorporated to enrich the searching behaviors and to avoid premature convergence, and an effective adaptive meta-Lamarckian learning strategy is employed to decide which neighborhood to be used. In addition, a pairwise based local search is applied after the SA-based local search to further enhance the exploitation ability.

The experimental results are listed in Table 2. The results of PSOMA come from [2] directly. In Table 2, *C* is the optimal makespan or lower bound value known so far, BRE represents the best relative error to *C*, ARE denotes the average relative error to *C*, WRE represents the worst relative error to *C*, and *T*_avg_ is the average running time (in second) of 20 independent runs. The boldfaces represent the better results in Table 2.

From Table 2, it is easy to see that the proposed algorithm obtained better solutions than those obtained by PSOMA for most of test problems considered (that is to say, the BRE values are very small). For those simple problems with smaller scales such as Car1~Car8, the proposed algorithm can obtain the global optimal value in 20 runs. For car5 and car6, the proposed algorithm can provide better average relative error and worst relative error than those obtained by PSOMA. For test problem Rec01~Rec11, the proposed algorithm is better than PSOMA for obtaining smaller ARE and WRE except Rec05, and the two algorithms obtained the same BRE. For test problems with 20 jobs and 15 machines, such as Rec13 and Rec17, the results of PSOMA were better than those of MPSOMA, while for the same type of test problem Rec15, BRE, ARE, and WRE values obtained by MPSOMA are better than those obtained by PSOMA. For Rec19~Rec21 with 30 jobs and 10 machines, the proposed algorithm perform better than PSOMA whatever BRE, ARE, and WRE are. For the rest of the test problems with larger scale such as Rec25~Rec41, the proposed algorithm obtained better results or similar ones as to those obtained by PSOMA. However, we also see from Table 2 that the proposed algorithm took much running time than that of PSOMA; the reason lies in three local search mechanisms which were performed on each individual in the current swarm.

### 4.2. Comparisons of PSOEDA and MPSOMA

To give a further evaluation on the performance of our proposed algorithm, we compare the proposed algorithm with a hybrid particle swarm optimization with estimation of distribution algorithm (PSOEDA) [22], which is recently proposed algorithm for PFSSP. PSOEDA is based on the discrete PSO and a new information sharing mechanism in swarm is proposed which not only comprise the experience of the global best solution and of the personal best solutions, but also of the information from the collective experience of all the particles. The new information sharing mechanism is converted from the EDA [24]. Some local search algorithms are also proposed to enhance the solution. All results of PSOEDA are also selected from [22]. The experiments results are shown in Table 3. Reference [22] did not provide the average running time, so we did not compare the average running time of the two algorithms in Table 3.

From Table 3, we can see that for test problems Car1~Car8, PSOEDA and MPSOMA presented the same performance in 20 independent runs. For Rec01 and Rec03, the proposed algorithm is better than PSOEDA on ARE and WRE, and both algorithms obtained the same results on Rec05. For the test problems Rec07~Rec11 with 20 jobs and 10 machines, the performance of the proposed algorithm is better than that of PSOEDA and provides a better BRE and WRE. However, for the test problems Rec13~Rec17, PSOEDA can obtain better results than MPSOMA. For Rec19~Rec29 with 30 jobs, the proposed algorithm can obtain better or same results as those of PSOEDA. For the test problems with large scale, the proposed algorithm provided competitive result.

### 4.3. The Results of Wilcoxon Matched-Pairs Signed-Rank Test

In order to illustrate the difference between the proposed algorithm and other contrastive algorithms such as PSOMA and PSOEDA, we use the Wilcoxon matched-pairs signed-rank test [28] on the optimal makespan results obtained by 20 independent runs for several test problems. The Wilcoxon Matched-Pairs Ranks test is a nonparametric test that is often regarded as being similar to a matched pair *t*-test and it is used to determine differences between groups of paired data when the data do not meet the rigor associated with a parametric test. The statistic results are provided in Table 4.

For Car7, Car8, Rec03, and Rec05, there is no difference among MPSOMA, PSOMA, and PSOEDA on the optimal makespan results, which means the proposed algorithm presented same results as to those of the other two algorithms on simple test problems. For Rec07~Rec25 with 20 jobs, the differences between MPSOMA and PSOMA are significant with *P* < 0.05; for the rest of test problems with 30 jobs, the differences between MPSOMA and PSOMA are still significant. For MPSOMA and PSODEA, the differences are significant for most of test problems except Rec15, Rec17, and Rec23. The results of the three test problems obtained by comparing MPSOMA and PSODEA cannot reject *H*_0_; that is to say, there are no differences between them. The results of statistical tests confirm that MPSOMA is the best one among all the tested algorithms for most of test problems considered in this study.

### 4.4. The Usefulness of Different Local Search Schemes Employed in MPSOMA

In order to illustrate the usefulness of three local search such as SA, IIS, and VNS used in the proposed algorithm, we will execute an extensive experiment on some test problems employed in this study.

Firstly, we removed the SA local search from the MPSOMA, namely, in the procedure of MPSOMA given in Algorithm 1; step 9 is skipped; we denote the algorithm without SA local search as MPSOMA-no-SA, and the performance of MPSOMA is compared with that of MPSOMA-no-SA on the test problems used in Section 4.1. Each test problem performs 20 independent runs and record ARE, BRE, WRE, and *T*_avg_. The experimental results are presented in Table 5.

It is easy to see that MPSOMA are better than MPSOMA-no-SA on most of test problems. For those simple problems such as Car1~Car8, the two algorithms can obtain the global optimal value in 20 runs except Car5. For Rec01~Rec07, MPSOMA showed a small advantage and had a better stability since it obtained better ARE. For rest of test problem, the performance of MPSOMA is superior to that of MPSOMA-no-SA. From the view of average running time, MPSOMA-no-SA without SA local search showed a little advantage over MPSOMA. We can conclude that SA local search is very important contribution on improving the performance of the proposed algorithm. The large running time taken by MPSOMA is mainly expended on two other local searches, IIS and VNS, since they are performed on all individuals in swarm other than the best one.

Secondly, we will explain why we used IIS on the odd subpopulations. Here, it is pointed out that three subpopulations are equal no matter which local search used in our study is performed arbitrary subpopulation. In the following, we will set such experiments: three subpopulations are updated by using the same local search (IIS or VNS) in step 4 or two subpopulations are updated by using VNS other than IIS like step 4 and the rest subpopulations is optimized by IIS. We performed on the first Car1~Rec17 to show the effect of different experimental settings.  Table 6 showed the statistic results under the same parameters setting as Section 4.1, in which two VNSs and one IIS indicates that there are two subpopulations that are updated by VNS and another subpopulation is adopted IIS in the procure of MPSOMA. Three VNSs and three IISs mean that three subpopulations are updated by using VNS or IIS.

From Table 6, we can see that for test problems Car1~Car8, all of the three algorithms with different local search setting can obtains the global optimal value in 20 runs, since BRE, ARE, and WRE resulted from them are all zeros. For most of the rest of test problems, the MPSOMA can provide better ARE and WRE. From this set of experimental results, we can see that MPSOMA with two IISs local search and one VNS local search showed a better performance than that by the other three experimental settings.

## 5. Conclusion

In this paper, we have attempted to enhance the performance of MA for PFSSP by introducing multipopulations cooperative coevolution and proposed a multipopulation particle swarm optimization (PSO) based memetic algorithm (MPSOMA). Each subpopulation is evolved by the global search mechanism, namely, the standard PSO, and then all particles in three subpopulations are updated by using different local search schemes with different probabilities which can produce offspring subpopulations with large distinction. After that, a multipopulation cooperative coevolution is realized by EDA and the information of the best particles from the different subpopulations is exchanged among these subpopulations so as to boost the proposed algorithm to find optimal solution for PFSSP. Experimental results on 26 benchmarks show that the proposed algorithm are competitive. However, the running time become large with the increasing number of job and the main reason lies in that local search is executed on all particles in three subpopulations. How to solve this problem is planned for our future work.

## Figures and Tables

**Algorithm 1:**
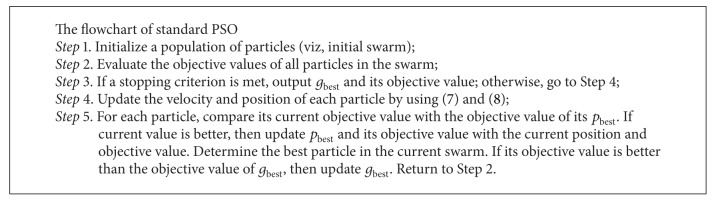
The flowchart of standard PSO.

**Algorithm 2:**
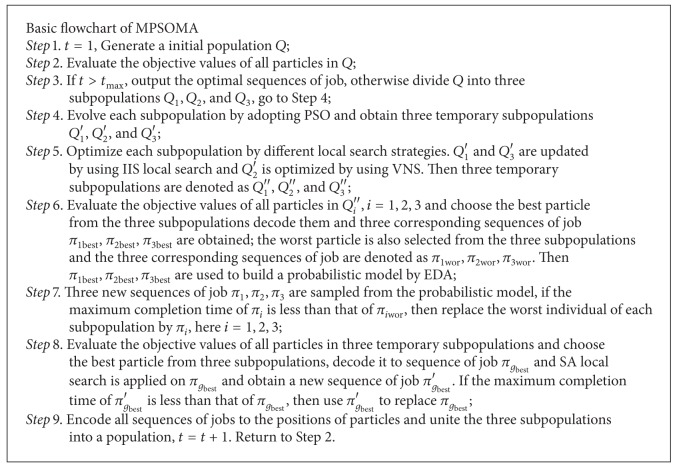
The flowchart of MPSOMA.

**Algorithm 3:**
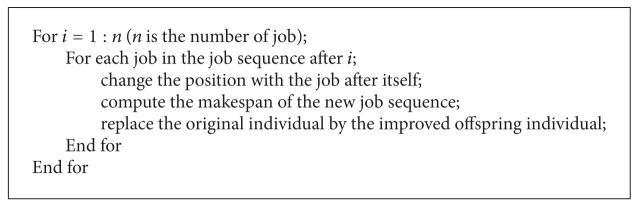
The basic flowchart of IIS local search.

**Algorithm 4:**
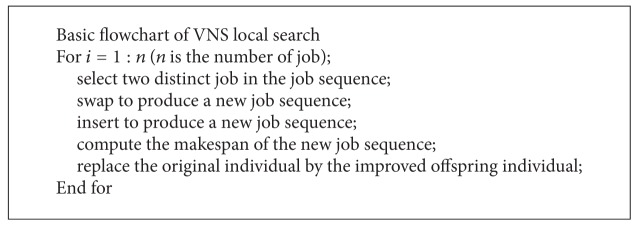
The basic flowchart of IIS local search.

**Algorithm 5:**
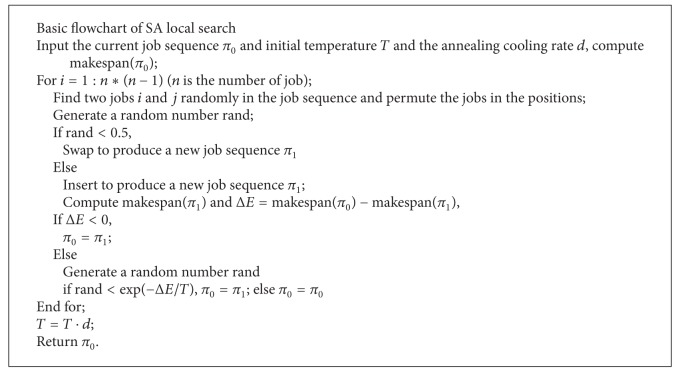
The basic flowchart of SA local search.

**Table 1:** The example of ROV rule.

Dimension *j*	1	2	3	4	5	6
Position value	0.6	2.9	1.8	3.7	2.1	0.7
Ranked-order value	1	5	3	6	4	2

**Table 2:** Comparisons of PSOMA and MPSOMA.

Problem	*n*, *m*	*C*	PSOMA				MPSOMA			
			BRE	ARE	WRE	*T*_avg_ (s)	BRE	ARE	WRE	*T*_avg_ (s)
Car1	11, 5	7038	0	0	0	0.68	0	0	0	24.35
Car2	13, 4	7166	0	0	0	0.95	0	0	0	40.36
Car3	12, 5	7312	0	0	0	1.06	0	0	0	32.31
Car4	14, 4	8003	0	0	0	1.22	0	0	0	53.13
Car5	10, 6	7720	0	0.018	0.375	0.70	0	**0**	**0**	22.10
Car6	8, 9	8505	0	0.114	0.764	0.49	0	**0**	**0**	17.52
Car7	7, 7	6590	0	0	0	0.30	0	**0**	**0**	8.09
Car8	8, 8	8366	0	0	0	0.42	0	**0**	**0**	11.47
Rec01	20, 5	1247	0	0.144	0.160	2.60	0	**0**	**0**	240.91
Rec03	20, 5	1109	0	0.189	0.721	2.50	0	**0**	**0**	231.94
Rec05	20, 5	1242	0.242	0.242	0.242	2.39	0.242	0.242	0.242	216.64
Rec07	20, 10	1566	0	0.986	1.149	2.81	0	**0**	**0**	337.90
Rec09	20, 10	1537	0	0.691	1.041	4.23	0	**0**	**0**	329.79
Rec11	20, 10	1431	0	0.129	0.978	3.79	0	**0**	**0**	329.21
Rec13	20, 15	1930	0.259	0.893	1.502	4.64	**0.104**	**0.891**	**1.192**	440.11
Rec15	20, 15	1950	**0.051**	**0.628**	**1.076**	5.23	0.615	0.877	1.333	455.37
Rec17	20, 15	1902	**0**	**1.330**	**2.155**	4.67	1.419	1.719	2.103	447.49
Rec19	30, 10	2093	0.43	1.313	2.102	10.49	**0.287**	**0.506**	**0.860**	1.66*e* + 03
Rec21	30, 10	2017	1.438	1.596	1.636	8.41	**0.992**	**1.393**	**1.438**	1.69*e* + 03
Rec23	30, 10	2011	0.596	1.310	2.038	9.36	**0.398**	**0.552**	**0.796**	1.66*e* + 03
Rec25	30, 15	2513	0.835	2.085	3.223	12.64	**0.279**	**1.337**	**1.989**	2.46*e* + 03
Rec27	30, 15	2373	1.348	1.605	2.402	12.15	**0.969**	**1.336**	**1.728**	2.25*e* + 03
Rec29	30, 15	2287	1.442	1.888	2.492	11.31	**0.235**	**0.969**	**1.547**	2.17*e* + 03
Rec31	50, 10	3045	1.510	2.254	2.692	37.15	**0.699**	**1.237**	**1.792**	1.55*e* + 04
Rec33	50, 10	3114	0	0.645	0.835	36.07	0	**0.326**	**0.835**	1.47*e* + 04
Rec35	50, 10	3277	0	0	0	29.92	0	0	0	1.32*e* + 04
Rec37	75, 20	3045	2.101	3.537	4.039	170.2	**1.696**	**2.450**	**3.827**	1.98*e* + 05
Rec39	75, 20	3114	**1.553**	2.426	2.830	155.7	**0.581**	**1.298**	**1.792**	1.67*e* + 05
Rec41	75, 20	3277	2.641	3.684	4.052	164.3	**1.890**	**2.428**	**2.282**	1.82*e* + 05

**Table 3:** Comparisons of PSOEDA and MPSOMA.

Problem	*n*, *m*	*C*	PSOEDA			MPSOMA		
			BRE	ARE	WRE	BRE	ARE	WRE
Car1	11, 5	7038	0	0	0	0	0	0
Car2	13, 4	7166	0	0	0	0	0	0
Car3	12, 5	7312	0	0	0	0	0	0
Car4	14, 4	8003	0	0	0	0	0	0
Car5	10, 6	7720	0	0	0	0	0	0
Car6	8, 9	8505	0	0	0	0	0	0
Car7	7, 7	6590	0	0	0	0	0	0
Car8	8, 8	8366	0	0	0	0	0	0
Rec01	20, 5	1247	0	0.096	0.160	0	**0**	**0**
Rec03	20, 5	1109	0	0.036	0.180	0	**0**	**0**
Rec05	20, 5	1242	0.242	0.242	0.242	0.242	0.242	0.242
Rec07	20, 10	1566	0	0	0	0	0	0
Rec09	20, 10	1537	0	0.202	1.041	0	**0**	**0**
Rec11	20, 10	1431	0	0.126	0.629	0	**0**	**0**
Rec13	20, 15	1930	0.104	**0.223**	**0.415**	0.104	0.891	1.192
Rec15	20, 15	1950	**0**	**0.303**	**0.667**	0.615	0.877	1.333
Rec17	20, 15	1902	**0**	**0.289**	**0.999**	1.419	1.719	2.103
Rec19	30, 10	2093	0.287	0.612	1.003	0.287	**0.506**	**0.860**
Rec21	30, 10	2017	1.140	1.408	1.438	**0.992**	**1.393**	**1.438**
Rec23	30, 10	2011	0.398	0.597	1.840	0.398	**0.552**	**0.796**
Rec25	30, 15	2513	0.279	1.894	2.507	0.279	**1.337**	**1.989**
Rec27	30, 15	2373	0.969	1.584	2.507	0.969	**1.336**	**1.728**
Rec29	30, 15	2287	0.350	1.045	1.618	**0.235**	**0.969**	**1.547**
Rec31	50, 10	3045	**0.263**	**0.430**	**0.657**	0.699	1.237	1.792
Rec33	50, 10	3114	0	**0.469**	0.835	0	**0.326**	0.835
Rec35	50, 10	3277	0	0	0	0	0	0
Rec37	75, 20	3045	1.838	2.725	4.04	**1.696**	**2.450**	**3.827**
Rec39	75, 20	3114	0.924	1.409	**1.730**	**0.581**	**1.298**	1.792
Rec41	75, 20	3277	**1.815**	2.506	2.940	1.890	**2.428**	**2.282**

**Table 4:** The results of Wilcoxon matched-pairs signed-rank test of MPSOMA with PSOMA and PSOEDA.

Problem	*n*, *m*	*C*	v.s. PSOMA	v.s. PSOEDA
Car6	8, 9	8505	−**2.1180**	−1.1010
Car7	7, 7	6590	−1.3127	−1.2423
Car8	8, 8	8366	−1.3086	−1.2126
Rec03	20, 5	1109	−1.1461	−1.1461
Rec05	20, 5	1242	1.5000	−1.129
Rec07	20, 10	1566	−**1.9852**	−1.2252
Rec09	20, 10	1537	−**2.7304**	−**2.6731**
Rec11	20, 10	1431	−**3.4105**	−**3.0886**
Rec13	20, 15	1930	−**3.9106**	−**2.5051**
Rec15	20, 15	1950	−**2.4497**	−1.6440
Rec17	20, 15	1902	−**3.7239**	−0.0101
Rec19	30, 10	2093	−**1.9846**	−**2.3642**
Rec21	30, 10	2017	−**1.9652**	−**2.1660**
Rec23	30, 10	2011	−**2.2234**	−1.9051
Rec25	30, 15	2513	−**3.0483**	−**2.0333**

**Table 5:** Comparisons of MPSOMA-no-SA and MPSOMA.

Problem	*n*, *m*	*C*	MPSOMA-no-SA				MPSOMA			
			BRE	ARE	WRE	*T*_avg_ (s)	BRE	ARE	WRE	*T*_avg_ (s)
Car1	11, 5	7038	0	0	0	18.60	0	0	0	24.35
Car2	13, 4	7166	0	0	0	32.60	0	0	0	40.36
Car3	12, 5	7312	0	0	0	25.33	0	0	0	32.31
Car4	14, 4	8003	0	0	0	41.15	0	0	0	53.13
Car5	10, 6	7720	0	0.017	0.155	14.80	0	0	0	22.10
Car6	8, 9	8505	0	0	0	8.078	0	0	0	17.52
Car7	7, 7	6590	0	0	0	8.077	0	0	0	8.09
Car8	8, 8	8366	0	0	0	8.078	0	0	0	11.47
Rec01	20, 5	1247	0.160	0.160	0.160	204.42	**0**	**0**	**0**	240.91
Rec03	20, 5	1109	0	0.063	0.180	199.23	0	**0**	**0**	231.94
Rec05	20, 5	1242	0.242	0.242	0.242	190.77	0.242	0.242	0.242	216.64
Rec07	20, 10	1566	0	0.706	1.149	303.58	0	**0**	**0**	337.90
Rec09	20, 10	1537	0	0.293	0.911	298.34	0	**0**	**0**	329.79
Rec11	20, 10	1431	0	0.486	0.978	300.91	0	**0**	**0**	329.21
Rec13	20, 15	1930	0.777	1.324	2.073	385.50	**0.104**	**0.891**	**1.192**	440.11
Rec15	20, 15	1950	0.718	1.336	2.051	394.47	**0.615**	**0.877**	**1.333**	455.37
Rec17	20, 15	1902	**1.157**	2.224	3.155	391.88	1.419	**1.719**	**2.103**	447.49
Rec19	30, 10	2093	1.290	1.636	2.485	1.62*e* + 03	**0.287**	**0.506**	**0.860**	1.66*e* + 03
Rec21	30, 10	2017	1.438	1.596	1.636	1.75*e* + 03	**0.992**	**1.393**	**1.438**	1.69*e* + 03
Rec23	30, 10	2011	0.547	1.342	1.790	1.65*e* + 03	**0.398**	**0.552**	**0.796**	1.66*e* + 03
Rec25	30, 15	2513	1.990	1.026	3.263	2.33*e* + 03	**0.279**	**1.337**	**1.989**	2.46*e* + 03
Rec27	30, 15	2373	1.264	1.846	2.444	2.44*e* + 03	0.969	**1.336**	**1.728**	2.25*e* + 03
Rec29	30, 15	2287	1.705	2.392	3.017	2.08*e* + 03	**0.235**	**0.969**	**1.547**	2.17*e* + 03
Rec31	50, 10	3045	2.594	2.667	2.890	3.34*e* + 03	**0.699**	**1.237**	**1.792**	1.55*e* + 04
Rec33	50, 10	3114	0.610	0.822	1.124	1.20*e* + 04	**0**	**0.326**	**0.835**	1.57*e* + 04

**Table 6:** Comparisons of usefulness of different local search settings.

Problem	Two IISs and one VNS (MPSOMA)			Two VNSs and one IIS			Three VNSs			Three IISs		
	BRE	ARE	WRE	BRE	ARE	WRE	BRE	ARE	WRE	BRE	ARE	WRE
Car1	0	0	0	0	0	0	0	0	0	0	0	0
Car2	0	0	0	0	0	0	0	0	0	0	0	0
Car3	0	0	0	0	0	0	0	0	0	0	0	0
Car4	0	0	0	0	0	0	0	0	0	0	0	0
Car5	0	0	0	0	0	0	0	0	0	0	0	0
Car6	0	0	0	0	0	0	0	0	0	0	0	0
Car7	0	0	0	0	0	0	0	0	0	0	0	0
Car8	0	0	0	0	0	0	0	0	0	0	0	0
Rec01	**0**	**0**	**0**	0	0.128	0.160	0	0.128	0.160	0	0.144	0.160
Rec03	0	**0**	**0**	0	**0**	**0**	0	**0**	**0**	0	0.018	0.180
Rec05	0.242	0.242	0.242	0.242	0.242	0.242	0	**0.217**	0.242	0	0.230	0.242
Rec07	0	**0**	**0**	0	0.115	0.150	0	**0**	**0**	0	0.581	0.150
Rec09	0	**0**	**0**	0	0.020	0.130	0	0.016	0.195	0	0.5303	1.301
Rec11	0	**0**	**0**	0	0.090	0.489	0	0.035	0.345	0	1.013	2.865
Rec13	**0.104**	**0.891**	**1.192**	0.518	0.992	1.347	0.155	0.929	1.347	0.933	1.433	2.228
Rec15	**0.615**	**0.877**	**1.333**	0.664	0.918	1.336	0.761	0.892	1.482	0.701	0.918	1.641
Rec17	1.419	**1.719**	**2.103**	**0**	1.754	2.576	0.368	1.809	2.261	1.052	2.327	3.155
